# Vulnerability and resilience to Alzheimer’s disease: early life conditions modulate neuropathology and determine cognitive reserve

**DOI:** 10.1186/s13195-018-0422-7

**Published:** 2018-09-19

**Authors:** Sylvie L. Lesuis, Lianne Hoeijmakers, Aniko Korosi, Susanne R. de Rooij, Dick F. Swaab, Helmut W. Kessels, Paul J. Lucassen, Harm J. Krugers

**Affiliations:** 1 Brain Plasticity Group, SILS-CNS, University of Amsterdam, Science Park 904, 1098 XH Amsterdam, The Netherlands; 2 Department of Clinical Epidemiology, Biostatistics & Bio informatics, Academic Medical Centre, University of Amsterdam, Meibergdreef 9, 1105 AZ Amsterdam, the Netherlands; 3 The Netherlands Institute for Neuroscience, an Institute of the Royal Netherlands Academy of Arts and Sciences, KNAW, Meibergdreef 47, 1105 BA Amsterdam, The Netherlands; 4 Department of Cellular and Computational Neuroscience, SILS-CNS, University of Amsterdam, Science Park 904, 1098 XH Amsterdam, The Netherlands

**Keywords:** Resilience, Vulnerability, Early life, Stress, Neonatal handling, Hypothalamic–pituitary–adrenal axis, EGR1, REST, Arc, Aging

## Abstract

**Background:**

Alzheimer’s disease (AD) is a progressive neurodegenerative disorder with a high prevalence among the elderly and a huge personal and societal impact. Recent epidemiological studies have indicated that the incidence and age of onset of sporadic AD can be modified by lifestyle factors such as education, exercise, and (early) stress exposure. Early life adversity is known to promote cognitive decline at a later age and to accelerate aging, which are both primary risk factors for AD. In rodent models, exposure to ‘negative’ or ‘positive’ early life experiences was recently found to modulate various measures of AD neuropathology, such as amyloid-beta levels and cognition at later ages. Although there is emerging interest in understanding whether experiences during early postnatal life also modulate AD risk in humans, the mechanisms and possible substrates underlying these long-lasting effects remain elusive.

**Methods:**

We review literature and discuss the role of early life experiences in determining later age and AD-related processes from a brain and cognitive ‘reserve’ perspective. We focus on rodent studies and the identification of possible early determinants of later AD vulnerability or resilience in relation to early life adversity/enrichment.

**Results:**

Potential substrates and mediators of early life experiences that may influence the development of AD pathology and cognitive decline are: programming of the hypothalamic–pituitary–adrenal axis, priming of the neuroinflammatory response, dendritic and synaptic complexity and function, overall brain plasticity, and proteins such as early growth response protein 1 (EGR1), activity regulated cytoskeleton-associated protein (Arc), and repressor element-1 silencing transcription factor (REST).

**Conclusions:**

We conclude from these rodent studies that the early postnatal period is an important and sensitive phase that influences the vulnerability to develop AD pathology. Yet translational studies are required to investigate whether early life experiences also modify AD development in human studies, and whether similar molecular mediators can be identified in the sensitivity to develop AD in humans.

## Background

Alzheimer’s disease (AD) is a neurodegenerative disorder that is highly prevalent among the elderly population. AD is characterised by progressive impairments in various behavioural and cognitive functions [1] that have a profound impact on AD patients, their families, caregivers, and society. Prominent neuropathological hallmarks in the brains of AD patients include amyloid-beta (Aβ) peptide-containing plaques and neurofibrillary tangles (NFTs) containing hyperphosphorylated tau. In humans and rodents, the gradual accumulation of Aβ-containing plaques and NFTs has been associated, among other things, with spine loss and glial activation. Together, they may trigger the age-related cognitive decline and behavioural symptoms characteristic of AD [2]. Seminal genetic studies have identified mutations in the amyloid precursor protein (APP), Presenilin-1, and Presenilin-2 genes and variations in ApoE in relation to early and late-onset familial AD (see e.g. [3–5]). While these mutations explain a small percentage of AD cases, the vast majority of cases probably have a multifactorial aetiology, in which both age and lifestyle factors play an important modulatory role [4, 6–8]. Epidemiological studies have shown that factors like higher education, a more healthy diet, more social and physical activities, bilingualism, and measures for lifelong learning and mental stimulation correlate with a slower rate of memory decline during aging, a delayed onset of mild cognitive impairment (MCI), and/or a lower incidence of AD [9–18]. These positive lifestyle factors may therefore be related to delayed AD onset and increase the resilience to develop AD.

On the other hand, adverse environmental experiences such as prolonged exposure to stressful experiences have been associated with a faster progression of AD symptoms and an earlier development of pathology [19, 20]. Stressful life events have been reported to reduce the age of onset in familial AD [19], while major depression, which has a strong stress-related component, has been associated with an increased risk to develop AD earlier in life (e.g. [19, 21]). Furthermore, glucocorticoid (GC) hormones, the main mediators of the stress response, are often found to be increased in AD, notably already in early phases of the disease [22–26]. Finally, dysregulation of the hypothalamus–pituitary–adrenal (HPA) axis (i.e. the main neuroendocrine axis controlling GC release and feedback) may increase the risk to develop AD [21, 23, 27]. Together, these studies highlight a possible interaction between genetic predisposition and lifestyle factors such as stress and/or low socio-economic status in determining the vulnerability and resilience to develop AD.

In a recent study, Wang et al. [28] have identified the early life period (up until adolescence) in humans as a sensitive time window during which environmental factors can exert pronounced and lasting effects on the risk to develop AD. During this sensitive time window early in life, the brain is showing enormous growth and development. This period of postnatal development is also very sensitive to environmental factors that may interfere with the ongoing development of brain structure and function, and can thereby program brain function for life [29–36]. Indeed, stressful and traumatic experiences during the early life period have been strongly associated with an increased vulnerability to stressors, and compromised physical and mental health in later life, both in humans and rodents [29, 31, 37–40]. On the other hand, ‘positive’ or stimulating early life experiences in humans [28] and rodents [41] have been associated with an apparent resilience to later-life challenges and a better physical and mental health.

Here, we discuss recent literature on the role of early life experiences in driving AD pathology. While human studies underscore the clinical and societal relevance of this topic, we focus on animal studies. Such studies allow for examining causal relationships, underlying molecular and cellular mechanisms, and a better understanding of how early life experiences and genes interact to determine the vulnerability to develop AD pathology. The findings are discussed in the context of theories on ‘cognitive reserve’ and ‘brain reserve’ (see Box 1), which help to conceptualise why some individuals may be more prone to develop AD than others. Finally, we identify possible molecular mediators and define critical outstanding questions that will help improve our understanding of how the early postnatal period can modify the risk to develop Alzheimer’s disease.

## Methods

We review literature and discuss the role of early life experiences in determining later age and AD-related processes from a brain and cognitive ‘reserve’ perspective. We focus on rodent studies and the identification of possible early determinants of later AD vulnerability or resilience in relation to early life adversity/enrichment.

## Results

### Early life experiences affect AD neuropathology and cognition

#### Early life adversity and AD

Genetically modified mice allow for the modelling of specific pathological features of AD such as Aβ and tau pathology (see Box 2 for an overview). Many studies in these mice have demonstrated effects of early life experiences on later cognitive function (see Box 3 for an overview of animal models of early life experiences). In the widely used APPswe/PS1dE9 mice, cognitive performance at an adult age was generally impaired when the mice had been exposed to prenatal or early life stress. For instance, exposing these mice to repeated brief periods of restraint stress from embryonic day 1 to 7 resulted in impairments in object location memory at 6 months of age [42]. In addition, maternal separation attenuated spatial learning in the offspring as tested in the Morris water maze task in 9-month-old mice [43]. Furthermore, APPswe/PS1dE9 mice exposed to chronic early life stress from PND 2 to 9 displayed cognitive impairments 1 year later, specifically in cognitive flexibility [44]. These latter effects were not caused by early life stress alone, since wild-type mice exposed to early life stress were not impaired. This suggests that early life stress may accelerate and/or aggravate symptom development [44].

These cognitive impairments are often accompanied by alterations in Aβ neuropathology. In middle-aged APPswe/PS1dE9 mice, both the plaque load and the soluble intracellular Aβ levels were increased following early life stress exposure [43–45], although at 4 months of age also a decrease in cell-associated Aβ has been reported after early life stress [45]. Counterintuitively, exposure to prenatal restraint stress reduced the plaque load in the hippocampus of 7-month-old female APPswe/PS1dE9 mice when compared to control-reared female transgenic mice, while no effects were found on intracellular Aβ immunoreactivity [42]. These effects were also not observed in male offspring, which remained unaffected by prenatal stress exposure. Effects of early life adversity on later AD measures have been studied in other transgenic mouse models as well. For instance, in a model co-expressing mutant APP and tau (biAT-mice), chronic early life stress increased soluble Aβ levels already in 4-month-old mice and reduced life expectancy [46]. This illustrates that in a genetic background relevant for AD, additional exposure to early life stress can increase Aβ neuropathology prior to the onset of cognitive impairments and even affect life expectancy.

Interestingly, the effects of stress early in life on both later cognition and AD-related neuropathology might not be specific for transgenic animals. In wild-type rodents, impairments in cognition occur following maternal separation, and are accompanied by increased levels of Aβ40 and Aβ42, an exacerbation of Aβ pathology [47], BACE expression [48], and/or tau phosphorylation [47, 49–53]. While in wild-type animals the Aβ monomers do not aggregate into Aβ plaques, these findings suggest that, regardless of the genetic background of an animal, stress exposure, be it early or later in life, promotes APP processing towards the production of more amyloidogenic species, and may thereby modify the sensitivity to develop AD pathology later in life.

#### Early life enrichment and AD

Although less well studied, exposing mice to an enriched and ‘positive’ environment during the early life period exerts opposite effects on cognition and AD-related neuropathology compared to early life stress [41, 46, 54]. For instance, neonatal handling, twice daily from PND 1 to 21, which has been associated with enhancing levels of maternal care, prevented spatial cognitive deficits and emotional alterations at 4 months of age in 3xTg-AD mice, an effect that was most pronounced in females [54]. Similarly, daily handling from PND 2 to 9 prevented the cognitive impairments in APPswe/PS1dE9 mice at 11 months of age [41]. Interestingly, while deficits in hippocampus-dependent and prefrontal cortex (PFC)-dependent memory performance were prevented by the neonatal handling procedure in this study, amygdala-dependent memories were not affected [41]. In line with this, neonatal handling reduced the Aβ plaque load in the hippocampus, but not in the amygdala [41]. Finally, in 4-month-old biAT mice, neonatal handling reduced Aβ levels prior to the emergence of cognitive deficits, and prolonged life expectancy [46].

Together, these studies indicate that neonatal handling reduces or delays the incidence of AD-related pathology, although differential effects on hippocampal and amygdala function were reported. Possibly, the developmental time window during which environmental manipulations are applied may have different outcomes. So far, it remains elusive what defines the optimal time window for installing lasting protective effects, an area of research that deserves more attention. In addition to the effects of positive stimuli during the early life period there are other studies showing protective effects of environmental stimuli, such as housing mice in enriched environmental conditions or exercise at adult or late age, on cognitive or neuropathological measures in different AD models.

#### Conclusion: Early life experiences modulate AD neuropathology and cognition

There is substantial evidence from transgenic rodent studies which supports the concept that the perinatal environment determines the vulnerability or resilience for AD-related cognitive impairments and Aβ neuropathology later in life. Early life adversity generally worsens cognitive performance and aggravates Aβ neuropathology, while early life enrichment can delay these cognitive deficits, at least for some behavioural domains, and attenuates Aβ neuropathology.

### Direct pathways

There are multiple pathways that can mediate the effects of early life experiences on cognition and AD neuropathology. First of all, there are pathways that are affected by early life experiences, and that are known to directly affect either the production or clearance of Aβ. The steady-state levels of Aβ depend on a balance between APP processing, the rate of Aβ production, and clearance of the peptide from the brain [55]. Likewise, tau hyper-phosphorylation can also be potentiated by factors induced early in life.

#### Hypothalamus–pituitary–adrenal axis

The hypothalamus–pituitary–adrenal (HPA) axis controls circulating glucocorticoid hormones (cortisol in humans, corticosterone in rodents). In response to corticotrophin releasing hormone (CRH), the pituitary releases adrenocorticotropin hormone (ACTH), which in turn stimulates the release of glucocorticoid hormones from the adrenal cortex [56]. At the early stages of AD, basal levels of circulating cortisol are often elevated [26, 57–59]. AD and dementia patients also show a failure to suppress their endogenous cortisol after administration of the synthetic glucocorticoid dexamethasone [25, 60, 61], indicating a dysfunction in the feedback of the HPA axis. Elevated basal cerebrospinal fluid (CSF) cortisol levels were specifically found in MCI patients who later developed AD, but not in MCI patients with other underlying neuropathologies. Moreover, higher baseline CSF cortisol levels were associated with a faster clinical worsening and cognitive decline in the MCI patients who were developing AD [62]. However, HPA dysfunction does not seem to worsen any further as the disease progresses [63, 64], suggesting that early life-induced alterations in HPA axis function, possibly acting via glucocorticoids, may in particular contribute to the onset and acceleration of AD pathogenesis, after which a new balance in HPA axis activity is reached. Rodent studies further indicate that pharmacological treatment with (synthetic) glucocorticoids or repeated stress exposure can induce pathological processing of both Aβ and tau. Both stress-level glucocorticoid administration in 3xTg-AD mice [65] and stress induction in wild-type rats [66] increase the levels of APP and the β-APP cleaving enzyme 1 (BACE1), which in turn increases amyloidogenic processing of APP and results in elevated levels of APP-derived fragments (C99 and C83) and Aβ peptides.

The early life postnatal environment is a strong determinant of HPA axis activity and later-life sensitivity to stressors [67]. In rodents, positive early life experiences generally dampen HPA axis reactivity, resulting in lower CRH and glucocorticoid levels in response to a stressor, whereas early life adversity generally increases HPA axis reactivity [67, 68]. As a consequence, the subsequent, cumulative exposure to glucocorticoids and/or CRH in adult animals is often persistently enhanced by early life stress. The notion that elevated glucocorticoid levels can promote Aβ levels (see earlier) may point to a critical role for these hormones in moderating AD neuropathology after early life adversity [65, 69, 70].

This points to the possible involvement of glucocorticoids in the initial development, or later promotion, of AD neuropathology, rather than that the alterations in glucocorticoids observed in AD may result from disease progression. However, prolonged glucocorticoid exposure, or exposure after early life stress, most likely cannot fully account for the neuropathological effects observed. Following chronic early life stress, wild-type animals show decreased corticosterone levels in response to an acute stressor, whereas APPswe/PS1dE9 mice exposed to the same paradigm, but not control-reared AD mice, display elevated corticosterone levels [44]. Thus, AD neuropathology by itself can also affect HPA axis functioning, which may depend on disease severity.

Notably, early life stress also increases the expression of BACE1 in adult wild-type mice [47, 71, 72] and APPswe/PS1dE9 mice [44]. The enhanced BACE1 expression following early life or adult stress exposure can be a direct effect of altered glucocorticoid signalling, as BACE1 contains glucocorticoid binding sites [73]. Indeed, short-lasting treatment with the glucocorticoid receptor antagonist mifepristone rescued the early life stress-induced cognitive impairments in APPswe/PS1dE9 mice and reduced the Aβ load and BACE1 expression [44]. In addition, a reduction in APP-derived C99 and C83 fragments was reported in 3xTg-AD mice after a similar treatment [74]. This suggests that the same pathway was affected by both manipulations and hence that APP processing is specifically targeted by (anti)-glucocorticoid actions. Alternatively, it has also been suggested that epigenetic modifications are responsible for the enhanced BACE1 expression [75].

Besides glucocorticoids, other stress mediators (such as CRH) have also been implicated in AD-related neuropathology. AD patients display reduced levels of CRH in the cortex and CSF [76, 77]. Rodent studies have further identified a role for CRH in protecting neurons against Aβ-associated cell death [78], possibly by promoting non-amyloidogenic APP cleavage [79, 80]. In contrast to these findings is the observation that stress exposure elevated CRH levels as well as Aβ expression [81, 82]. The role of CRH in Aβ pathology therefore needs further investigation.

Although less extensively described in recent literature, chronic stress or glucocorticoid exposure also induces abnormal hyper-phosphorylation of tau in wild-type mice [50] and 3xTg-AD mice [65]. Glucocorticoids potentiate the ability of centrally infused Aβ to induce hyper-phosphorylation of tau epitopes associated with AD [50], suggesting that tau pathology is also affected by HPA axis-related mechanisms [83, 84]. Although speculative, this could be a mechanism by which early life experiences, via alterations in HPA axis activity, could modulate tau pathology. Together, these studies highlight the potential of alterations in glucocorticoids and CRH, both factors affected by early life experiences, to be involved in promoting AD pathology, and that modulating these systems may directly affect pathological markers such as Aβ production and tau hyper-phosphorylation. However, further research is warranted to understand the exact mechanisms how this occurs, and the causative nature of the effects, in particular regarding tau pathology.

#### Blood–brain barrier integrity

Aβ in the brain is controlled via a steady-state homeostatic balance of production and removal. In humans, approximately 25% of Aβ is cleared from the brain via the blood–brain barrier (BBB) [85]. Post-mortem studies have shown that BBB integrity declines with age [86, 87], and might be involved in the onset of dementia [88]. Both acute and chronic activations of the stress system may compromise the permeability of the blood–brain barrier [89, 90]. Restraint stress in rodents induces damage in the capillary brain endothelial cells and alters expression of the tight-junction proteins occludin, claudin-5, and glucose transporter-1 in these brain capillaries, pointing to impaired BBB functioning [90]. Interestingly, mice that are resistant to the induction of a depression-like phenotype after exposure to chronic social defeat stress (CSDS) showed an upregulation of claudin-5 levels and more intact brain endothelial cell morphology compared to mice sensitive to CSDS [89]. Although further experimental validation is required, particularly with regard to how early life experiences regulate BBB stability and permeability for life, (early) stress could possibly influence Aβ clearance from the brain through altering the permeability of the BBB.

#### Neuroinflammation

Another mechanism possibly involved in the clearance of Aβ from the brain is via the brain’s neuroinflammatory response. For example, microglia bind Aβ oligomers and fibrils and clear Aβ from the brain through the secretion of Aβ-degrading enzymes like neprilysin [91] and insulin-degrading enzyme (IDE) [92], and through the phagocytic uptake and active degradation of Aβ. Both IDE and neprilysin activities are reduced in AD, and, interestingly, are further inhibited by glucocorticoids [93]. In response to Aβ oligomers, microglia induce an acute inflammatory response to aid clearance and restore homeostasis [94–96]. In the prolonged presence of Aβ accumulation, however, the physiological functions of microglia, such as synaptic remodelling, are thought to be compromised and may lead to a chronic neuroinflammatory response [97]. This progressive microglial activation, elevated pro-inflammatory cytokine levels, and morphological changes of microglia may result in functional and structural alterations that ultimately can promote neuronal degeneration [97]. Adverse early life experiences have been reported to alter the number of microglial cells, their morphology, phagocytic activity, and gene expression in the developing hippocampus that extend into the juvenile period (reviewed in [98–100]). These changes in microglial function are associated with abnormalities in developmental processes known to be mediated by microglia, including synaptogenesis, synaptic pruning, axonal growth, and myelination (reviewed in [100, 101]), and make them more responsive to subsequent inflammatory challenges like Aβ (microglial ‘priming’) [99, 102–104]. Conversely, neonatal handling programmes the expression of the anti-inflammatory cytokine IL-10 early in development by decreasing its methylation within microglia, attenuating glial activation [105]. Recently, exposure to early life stress in APPswe/PS1dE9 mice was shown to increase the plaque load while attenuating microglial responses in a lasting manner [45]. Whether enhanced Aβ pathology reduces microglial response, or whether early life programming is truly causing alterations in microglial activation, which in turn may modulate Aβ neuropathology, requires further investigation.

Thus, impairments in glial functioning and/or in the inflammatory response to Aβ, possibly modulated or ‘primed’ by early life experiences, could lead to an altered Aβ phagocytic capacity or clearance, and hence an altered Aβ burden with increasing age. Further studies are required, both with regard to whether positive early life experiences increase AD resilience via the modulation of such neuroinflammatory responses, and regarding the extent to which, and how, early life events can indeed programme microglia directly and indirectly.

### Modulation of AD resilience/vulnerability through altered cognitive and brain reserve following early life experiences

Besides a direct modulatory role of early life experiences on AD neuropathology and related cognitive decline (i.e. via regulation of Aβ and tau), early life experiences may also modify the brain’s ability to cope with the pathological burden of AD. For instance, a healthier, more active, and more flexible brain may have a higher capacity to ‘circumvent’ or delay effects of an insult and hence cope in a better way with the challenges posed by the AD pathology [106]. This concept has been termed ‘brain reserve’ or ‘cognitive reserve’, and has been introduced to explain individual variation in vulnerability and resilience for age-related cognitive decline (see Box 1). These concepts stem from findings that brain pathology (such as plaque load) is an unreliable predictor of human cognitive performance given that, with a comparable pathological load, some patients perform better than others in cognitive tasks [107]. This could be a secondary mechanism, in addition to the mechanisms already described, through which early life experiences determine behavioural AD outcome.

#### Early life experiences, brain reserve, and cognitive reserve

The hypothesis that early life experiences influence brain or cognitive reserve, and may either protect against or aggravate the clinical consequences of AD neuropathology, comes from several epidemiological studies. For instance, individuals with less than 8 years of formal education had a 2.2 times higher risk of developing dementia than those with more than 8 years of education, and participants with low socio-economic status were at 2.25 times greater risk of developing dementia than those with high lifetime occupational attainment [108]. Conversely, higher education and higher social economic family status reduced the risk of dementia lastingly [28], while both the number of years of formal education [109] and higher school grades appear to protect against dementia, even in the absence of later-life educational or occupational stimulation [110]. Furthermore, elderly people participating in frequent leisure activities expressed a 38% lower risk of developing dementia [111]. Also, early life linguistic ability is a strong predictor for later-life cognitive performance and being raised in a bilingual family, for example, protects against AD [16, 112]. Conversely, the occurrence of a parental death between age 0 and 18 is associated with a higher incidence of AD [113]. Such associations between early life environmental factors and AD indicate that cognitive stimulation at an age at which the brain is still in development may contribute to the building of cognitive reserve and thereby reduce the risk for later AD, while disturbances like stress or trauma during early life can be detrimental for building cognitive reserve. This is consistent with observations in animal studies demonstrating the existence of specific ‘critical periods’ during early life [114, 115] when disturbances in neuroplasticity can have a long-lasting impact on brain function. Overall, these studies indicate that educational and possibly also specific occupational experiences may create a reserve that could delay effects of AD pathology. This does not exclude the important contribution of the genetic background nor of familial and societal factors that may also promote a higher educational and occupational stimulation and thereby a better coping with pathological load at later ages.

**Fig. 1:**
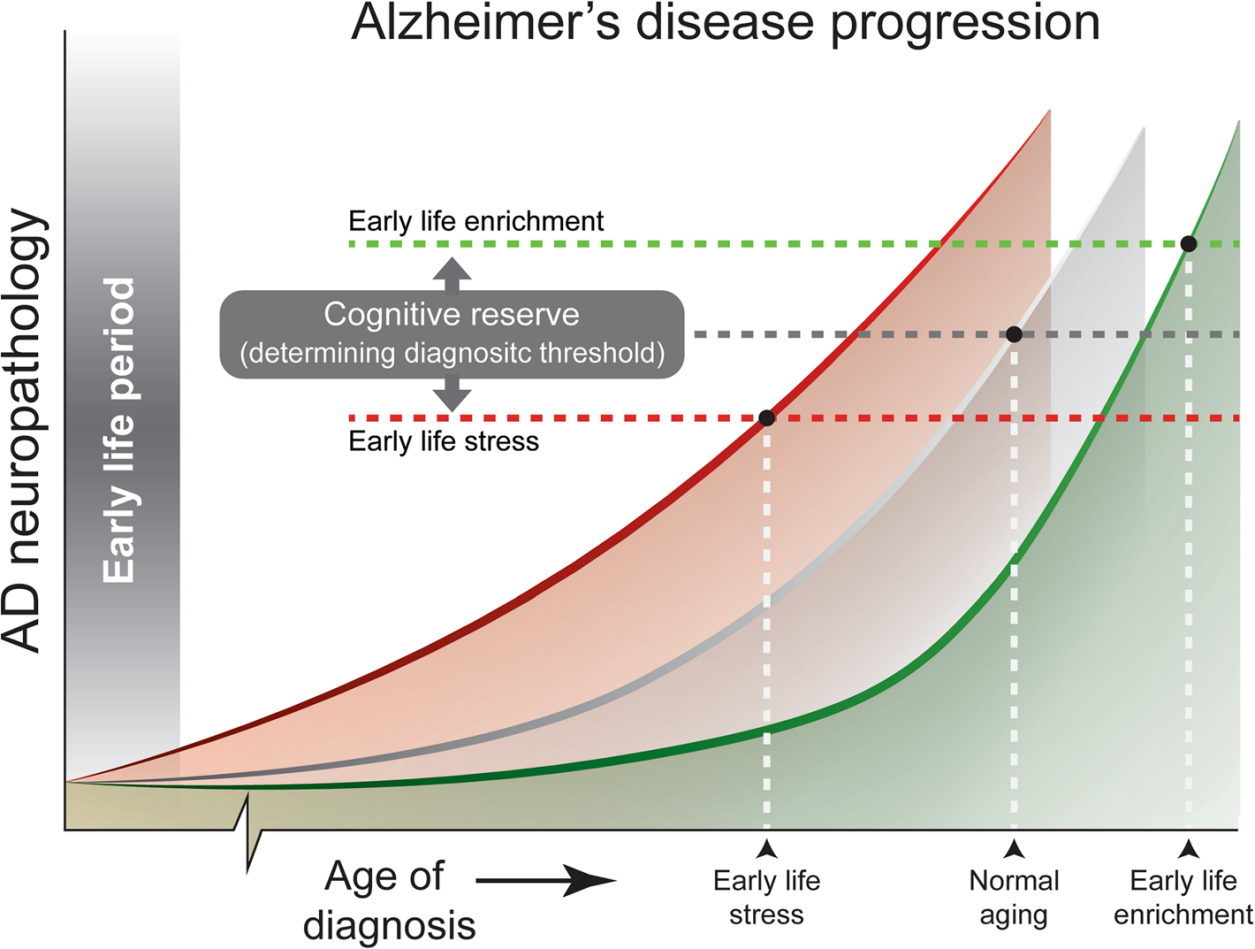
Proposed timelines illustrating how early life experiences can alter brain and cognitive reserve and impact development of AD neuropathology. Early life period determines rate at which AD neuropathology develops, with early life stress (red) accelerating disease progression, while early life enrichment (green) decreases disease progression. In addition, cognitive reserve of the brain is modulated by early life experiences, thereby determining at what pathological stage the clinical diagnosis of dementia is established. AD: Alzheimer’s disease

Although patients with high education/socio-economic status show a delayed onset of AD symptoms, they typically show a more rapid cognitive decline once the disease starts [116, 117]. This may suggest that patients with a high reserve can tolerate a higher burden of AD pathology in the brain, and that the time point at which cognitive functions begin to become affected will be later than in those with a lower cognitive reserve (Fig. 1). However, in all people, eventually a common point is reached when the pathology is so severe that brain function cannot be maintained anymore. Individuals with the greatest cognitive reserve will have a more advanced pathology at the onset of cognitive decline, although there will be less time until they reach the point when pathology overwhelms function, and then a more rapid rate of decline is expected [118] (Fig. 1).

Despite the support for this theory from epidemiological studies, more controlled studies aimed to determine whether (early) environmental factors can actually help build AD resilience are thus far lacking. In particular, the question remains open which molecular and cellular substrates mediate the effects of life experiences, especially those occurring early in life, on cognitive reserve and clinical AD outcome.

#### Animal research of early life experiences and cognitive reserve

Although attractive as a concept, it is currently unknown which brain mechanisms underlie brain and cognitive reserve. To address this, animal studies are required to address how a brain and cognitive reserve can be installed, and what the underlying molecular and cellular substrates are.

One possible mechanism for a cognitive reserve is the ability/capacity to compensate for dysfunction in one brain circuit by recruiting associated, unaffected brain circuitries, at least functionally. This would allow for switching between cognitive strategies and for using alternative and/or additional brain networks, to better cope with Aβ pathology. For instance, Granger et al. [119] observed that male and female mice overexpressing the human APP transgene exhibited similar neuropathological load. However, females displayed earlier cognitive impairments than males, which were able to compensate for Aβ-associated impairments by alternating navigational search strategies and by adopting increasingly productive spatial search strategies in the Morris water maze task. In contrast, females failed to efficiently switch from systematic to spatial learning strategies, potentially indicating a weaker cognitive reserve [119]. In addition, there is evidence that (early life) stress affects cognitive reserve. When presented with a dual-solution spatial navigation task, in which two different strategies can be employed to solve the task, both humans and mice, under a low stress condition, primarily use a hippocampus-dependent spatial strategy. However, when presented with a stressor prior to the task, they switch to a striatum-dependent stimulus-response strategy [120–125]. Likewise, prenatal and postnatal stress in rodents have been reported to bias navigation strategies towards more rigid, inflexible striatum-based learning strategies even under low stress conditions [126–128]. This indicates that early life stress decreases cognitive flexibility and the ability to activate different brain areas. This capacity for recruiting alternative strategies and related brain networks to solve problems has not been studied in relation to early life experiences and AD (although APPswe/PS1dE9 mice exposed to chronic early life stress show impaired behavioural flexibility, as measured by reversal learning on the Barnes maze [44]).

#### Mediators of early life experiences and brain reserve

Animal models have been used for detailed assessment of how early life experiences can affect components that may underlie brain reserve. This involves dendritic morphology, spine number, synaptic plasticity, and proteins that regulate synaptic function, which all determine plasticity of the brain and may render the brain more or less susceptible for AD-related pathological changes.

##### Dendritic morphology

Various studies have shown that prenatal and neonatal experiences cause persistent morphological changes in specific limbic brain regions and PFC [129–133]. For example, following early life stress, dendritic atrophy of CA1 pyramidal cells and expansions in the CA3 mossy fibres were observed, while the number of granule cells and the dendritic complexity in the hippocampal CA1 area and its innervation of CA3 pyramidal neurons were reduced [134], possibly via stress-induced increased CRH levels [68]. Furthermore, exposure to chronic early life stress reduced the number of dendritic spines, the anatomical substrate for memory storage and synaptic transmission, in both CA1 and CA3 areas and reduced inhibitory synaptic density in the CA1 area and excitatory synaptic density in the CA1 and CA3 areas of the hippocampus [135]. Although less well described, other brain regions are also affected, and chronic early life stress hampered dendritic development and spine density in the PFC [135, 136], while it increased spine density in the basolateral amygdala [137]. In addition, pups that received low amounts of maternal care early in life show reduced dendritic complexity in the CA1 area and dentate gyrus at adulthood, when, compared to pups that received high amounts of maternal care [34, 138, 139]. Also, the number of spines in hippocampal neurons was higher in pups that received high compared to low amounts of maternal care [138, 139]. Finally, maternal separation caused atrophy of the basal dendritic tree and reduces spine density on both the apical and basal dendrites in layer II/III of the PFC [140], and maternal deprivation reduced the number of granule cells and dendritic complexity in the dentate gyrus [141, 142], but had no effects in the amygdala [143]. These studies indicate that enhanced patterns of paternal sensitivity enhance dendritic complexity later in life in brain areas that are critical for learning and memory processes. This may therefore potentially affect cognitive function, synaptic plasticity (see below), and the cognitive reserve.

##### Synaptic plasticity

Disturbances in LTP have been implicated in the early manifestation of AD [144, 145]. Several in-vitro and in-vivo studies have directly implicated Aβ oligomers as a trigger of synaptic dysfunction (e.g. [146]), by weakening synapses, impairing LTP, and affecting the density of dendritic spines [145, 147–152]. Under conditions where LTP induction is already challenged—for example, following early life stress exposure [68, 134, 135, 138, 139, 142, 153–157]—the effects of Aβ on synapses and plasticity can be aggravated, thereby accelerating the onset of cognitive impairments. In contrast, when enhanced LTP is formed as a consequence of early life enrichment, the effects of Aβ can be alleviated, delaying the onset of cognitive impairments. As Aβ specifically targets synapses and disrupts synaptic signalling pathways, a larger or smaller dendritic tree and/or spine density could provide a structural substrate that could modulate effects of the first exposure to Aβ, and hence make specific synapses more or less vulnerable to Aβ-induced neuronal death. Together, alterations in synaptic plasticity, evoked by early life experiences, could influence the adult brain’s capacity to ‘circumvent’ AD-associated insults for a longer time, thus prolonging the period of healthy cognitive performance despite ongoing Aβ neuropathology.

##### Repressor element-1 silencing transcription factor (REST)

Recent studies have indicated how early life experiences can affect synaptic functions. For instance, during development there is a switch in NMDA-R composition, with GluN2B being predominantly present in the early postnatal brain. Over time, the number of GluN2A subunits increases, and after 2 weeks they outnumber the GluN2B [158]. This process can be disturbed by early life stress, as maternal deprivation slows down the switch to a mature, predominantly GluN2A-containing NMDA receptor phenotype at PND 28 to 31 [159]. Interestingly, by 8 weeks of age, the effects of early life stress on the GluN2B–GluN2A switch were reversed with more GluN2B expression in the hippocampus [156]. This disturbed developmental switch has been suggested to be mediated by an impaired activity of the transcriptional repressor REST in the hippocampus following early adversity [159]. REST is a gene-silencing factor expressed during development that inactivates neuronal genes important for synaptic functioning, among which is the gene encoding GluN2B, and is essential for the experience-dependent fine-tuning of gene expression involved in synaptic activity and plasticity [160, 161]. The composition of the NMDA receptor is of particular relevance as Aβ acts specifically via the GluN2B subunit, effecting a switch in subunit composition from GluN2B to GluN2A [162]. REST has been found to be present during normal aging of cortical and hippocampal cells but to be lost in both MCI and AD. Also, REST switches off genes promoting cell death while promoting the expression of various genes involved in the protection against stress [163]. Cognitively healthy elderly people indeed show increased REST levels compared to cognitively impaired elderly people. This makes REST an interesting candidate that could link early life experiences to later resilience to AD. However, whether changes in REST expression following early life experiences persist into aging remains to be further investigated.

##### Early growth response protein 1 (EGR1)

Another candidate to mediate effects of early life experiences on AD vulnerability/resilience is EGR1 (also commonly referred to as *Zif268*, NGFI-A, or KROX-24), a transcription factor critically involved in processes underlying neuronal activity, from neurotransmission and synaptic plasticity to higher order processes such as learning and memory, and to the response to emotional stress and reward [164–169]. EGR1 expression is induced in neurons by activity-dependent synaptic plasticity upon learning. Both the complete absence of and the heterozygous deletion of EGR1 are associated with impaired LTP maintenance over longer periods of time [170]. In contrast, EGR1 overexpression enhances LTP [171]. There is also extensive evidence that EGR1 expression is sensitive to natural environmental stimuli, such as learning tasks [172, 173], and learning-related increases in EGR1 expression have been reported in many paradigms and brain structures (e.g. [174, 175]).

EGR1 is expressed at low levels during the postnatal period. Over a period of about 2 weeks (for the hippocampus), expression levels slowly increase to reach adult levels [169]. Interestingly, neonatal handling increased EGR1 mRNA and protein levels [176], while postnatal restraint stress downregulated EGR1 [177]. Furthermore, early life stress induces rapid alterations in the acetylation of histones H3 and H4, which correlate with the expression of EGR1, and stress-induced activation of the GR itself also regulates EGR1 expression [178]. This highlights a role for EGR1 as an experience-dependent mediator of the adaptation to different early environments. It is tempting to speculate that the altered expression of EGR1, usually measured acutely after the early life period, may be a starting point for the long-term dendritic and synaptic reorganisation following these experiences.

EGR1 expression is of particular interest in shaping brain reserve in AD, as it is upregulated during the non-symptomatic stages of AD, but not in symptomatic stages in humans [179, 180], and is also downregulated in cognitively impaired aged mice [181, 182]. The effects of EGR1 may counteract Aβ-mediated synaptotoxicity; in patients who show AD pathology but do not have cognitive decline (Braak stages II–III), EGR1 may be upregulated to increase synaptic plasticity as an attempt to compensate for Aβ-induced neuropathology. After a certain threshold has been reached, EGR1 is no longer able to compensate sufficiently given the synaptotoxic consequences of Aβ, and cognitive impairment associated with the symptomatic stage of AD is thought to commence. Lower initial levels of EGR1 following early life adversity could thus result in a lower capacity to counteract, or ‘deal with’, Aβ neurotoxicity and an earlier display of cognitive impairment, whereas higher baseline EGR1 expression following positive early life experiences would allow the brain to counteract Aβ neurotoxicity for a longer period of time.

More recently, EGR1 has also been implicated as a driving factor of AD neuropathology and cognitive decline, since hippocampal EGR1 inhibition was shown to reduce tau phosphorylation, lower Aβ pathology, and improve cognition in 3xTG-AD mice [183]. Since EGR1 inhibition was also shown to activate BACE1 activity [184], this calls for further studies into the role of (early life) modulation of EGR1 and its implication in cognitive impairment and AD neuropathology.

##### Activity regulated cytoskeleton-associated protein (Arc)

Several potential target genes of EGR1 have been implicated in AD vulnerability, among which is the immediate-early gene Arc (also commonly referred to as Arg3.1), which is activated upon EGR1 expression [185, 186]. Arc is critical for memory consolidation [187] and is abundantly expressed in dendrites [188], the postsynaptic density [188], and the nucleus [189]. Glutamatergic neurons in the brain express Arc following increased synaptic activity associated with a range of behavioural and learning paradigms [190]. This process is altered in AD (models) [191–194]. Arc has been implicated in the homeostatic scaling of synaptic strength [195] by selectively lowering the levels of AMPA receptors that contain subunit GluA3 [196]. GluA3-containing AMPA receptors, in contrast to those containing subunit GluA1, traffic to synapses independent of neuronal activity [197, 198]. Thus, while active synapses are enriched for GluA1, synapses that are deprived of input are enriched for GluA3 [199]. Interestingly, the presence of GluA3 is required for Aβ to mediate synaptic and memory deficits [145], suggesting that Arc and GluA3 expression may render synapses resistant to Aβ. Besides this protective role, Arc may also contribute to the pathogenesis of AD by regulating the neuronal production of Aβ [194].

Arc expression is regulated via activation of GRs [200, 201], the expression of which is affected by early life experiences. Indeed, lifelong Arc expression can be determined early in life, and Arc mRNA expression was, for example, strongly reduced in aged rats with a history of maternal separation [71]. Furthermore, Arc expression is reduced with aging per se in wild-type animals [71], possibly underlying impairments in cognitive performance with older age, and particularly in AD. For example, following learning experiences, Arc expression was lower in the neocortex of AD transgenic mice, indicating an impairment in neuronal encoding and network activation [202]. Increased levels of Aβ in transgenic mice expressing human APP resulted in impaired Arc expression and hyperexcitable networks and the subsequent development of seizures [203, 204]. This suggests that increasing Arc levels prior to the development of AD neuropathology (e.g. through positive early life experiences) could possibly protect for a longer period of time against the cognitive impairments that accompany AD neuropathology.

#### Conclusion: early life environment and cognitive/brain reserve

Together, these findings highlight the programming role of early life experiences in specific measures reflecting brain and cognitive reserve. Dendritic morphology, spine density, synaptic protein expression, and the induction of LTP are all decreased/weakened following early life adversity, whereas a positive early life environment enhances/increases these parameters, resulting in later alterations in brain plasticity and behaviour. The installation of such alterations occurs prior to disease onset and can modify brain function at many levels. Consequently, these changes may determine the extent of reserve that the brain encompasses, and could determine its ability to later cope with further insults like the emergence of different aspects of AD neuropathology.

Experimental evidence for this hypothesis is thus far limited, and very few studies have addressed the effects of early life experiences on the aforementioned parameters in genetic AD models, while the preliminary studies published so far are not fully conclusive. Whether the molecular changes in, for example, REST, EGR1, and Arc expression following early life experiences indeed persist throughout the life span of an animal, and can thus actually affect the rate of aging, remains to be further investigated. A correct interpretation of the functional implications of the stress-induced or AD-induced upregulation or downregulation of some of these markers or processes underlying effects of early life experiences on cognitive reserve is further complex; the magnitude and direction of these neurochemical changes depend on a variety of factors, including the type and severity of the stressor, the age of the animal during stress exposure, and the age, sex, and species of the animal used upon testing, as well as the brain area and cell types studied. Further research is therefore needed to answer the question of whether the stress-induced upregulation or downregulation of a given process is beneficial or detrimental for neuronal and synaptic plasticity, and whether this may then mediate the potential to adapt brain and behaviour to a stressful or AD-related microenvironment before any clinical application of any of these targets can be implemented. In particular, carefully controlled, well-timed, and region-specific interventions on these targets in animal models should be performed before we can causally link them to AD resilience, let alone consider them as a target for human interventions.

## Discussion

### Lessons from animal models of AD

**Fig. 2:**
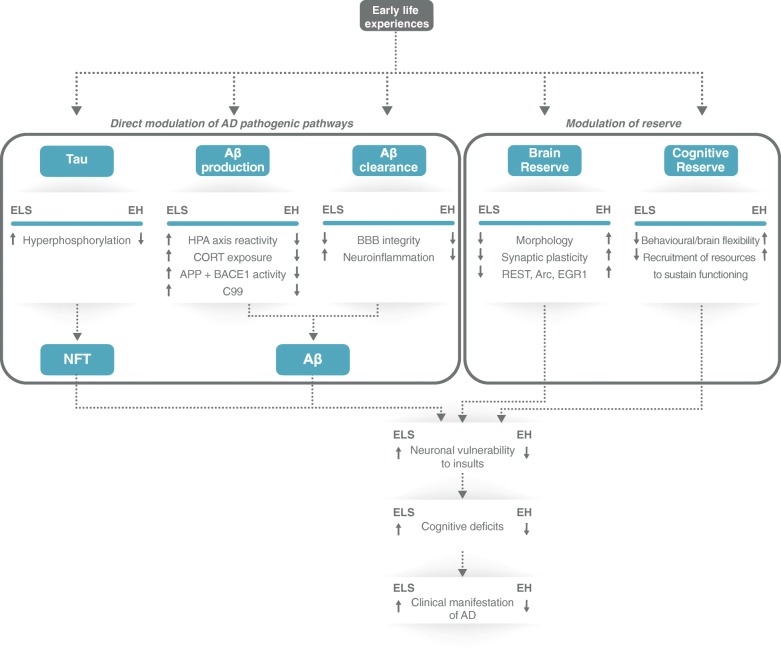
Model of how early life experiences could modulate later AD vulnerability or resilience. Early life experiences directly modulate AD pathogenic pathways by altering tau phosphorylation and production and clearance of Aβ, resulting in a higher pathological load. Secondly, early life experiences determine establishment of a cognitive and/or brain reserve, yielding the brain more vulnerable to pathological insults. Combined, these two pathways mediate effects of early life experiences on vulnerability or resilience of the brain to AD. Aβ amyloid beta, AD Alzheimer’s disease, APP amyloid precursor protein, Arc activity regulated cytoskeleton-associated protein, BACE1 β-APP cleaving enzyme 1, BBB blood–brain barrier, CORT corticosterone, ELS early life stress, EGR1 early growth response protein 1, HPA hypothalamic–pituitary–adrenal, REST repressor element-1 silencing transcription factor, EH early handling, NFT neurofibrillary tangles

In animal models for AD, early life experiences can have a profound impact on aging and survival, later cognitive function, and the development of AD-specific neuropathological features. These effects are two-sided: directly by altering disease-modifying factors, and/or indirectly by affecting the brain’s ability to cope with these insults. Early life experiences can determine the vulnerability or resilience to develop Alzheimer’s disease (Fig. 2) by persistently altering systems involved in both Aβ production and clearance. For instance, HPA axis hyperactivation after early life stress leads to cumulative increased exposure to glucocorticoids, which can directly (potentially) promote amyloidogenic processing of APP, potentially impair BBB integrity, and affect the neuroimmune response. Together, this may reduce the brain's clearance ability and enhance accumulation of Aβ in the brain. Conversely, early life ‘enrichment’ may lower lifelong glucocorticoid exposure and counteract these effects. Besides a direct modulation of the amyloidogenic processing, early life experiences may also programme the ability of the brain to cope with AD pathology. Positive and stimulating early life experiences can further increase factors associated with brain reserve such as dendritic and spine architecture, synaptic plasticity, and proteins such as EGR1, REST, and Arc. Consequently, this may influence the ability of the brain to cope with AD-related neuropathological changes before cognitive deficits become apparent. Conversely, early life stress can reduce these factors, making the brain less capable to cope with AD-related pathological changes. Although not yet addressed in sufficient detail, animal models for early life stress are particularly suitable to identify the so far unknown key molecular and cellular mechanisms that underlie brain and cognitive reserve and the correlations between specific early life experiences and later AD risk.

### Clinical implications

Identification of the factors that are causally related to AD resilience could be pivotal in individual risk assessment and determining disease vulnerability for aged individuals and MCI patients. In addition, these factors might aid the future development of early environmental and/or pharmacological interventions aimed to increase AD resilience (see Box 4 for an overview of the remaining outstanding questions). However, we warrant caution in the (over)interpretation of the available preclinical findings and their relevance in the clinic since the fundamental basis of the described targets and their causal relevance to AD is not yet fully understood, and the gap between preclinical and clinical studies can be vast. To bridge this gap, further clinical validation of the concepts identified in rodent studies may yield insight into their relevance for patients. In particular, existing longitudinal cohort studies could help identify first hints as to whether early stress affects AD-related parameters, and from there could help to identify critical time windows during which cognitive reserve is most effectively established. Cohort studies in which people have been followed into older age and in which data have been collected throughout life are specifically suitable, as this allows examination of the association between early life factors and prevalence of MCI and dementia as well as pre-symptomatic markers. For example, studies in the Dutch famine birth cohort have shown that exposure to malnutrition in early gestation, a severe early life stressor, was associated with poorer cognitive function in subjects with the age of 58 years, as well as smaller brain volumes and increased symptoms of brain ageing in men at age 68 years [205–207]. Alternatively, this could be further simplified and cohorts stratified when reliable ‘signatures’ or biomarkers of early life stress could be developed and would be available, as is now done for adult stress exposure based on hair cortisol measurements [208]. Furthermore, some of the molecular targets highlighted in this study that mediate effects of early life experiences on reserve are also modulated by learning processes per se. Thus, pharmacological interventions using these targets in the clinic are still far away, as many of these targets need to be further validated first, also due to their versatile functions and the expected accompanying side-effects. Moreover, these target proteins may also be influenced using environmental stimuli at older ages.

One of the few interventions that have been shown to be successful in rodent studies at older ages and after a relatively short treatment, while also being FDA approved, is targeting glucocorticoid hormones [44, 74]. Also, a small clinical trial in AD patients and old macaque monkeys reported improvements in cognition after treatment with mifepristone (GR antagonist) [209, 210], although the short time window and small sample size warrant caution in interpreting these results. Furthermore, AD patients with the highest baseline cortisol levels benefited most from a mifepristone intervention and showed persistent memory improvements up to 8 weeks after discontinuation of the treatment [210]. This could therefore potentially present a promising strategy to further explore, specifically in stress-enhanced AD presentation.

## Conclusion

The mechanisms identified through preclinical studies, supported by possible follow-up in validation studies to clinical pilot studies eventually, will hopefully benefit the identification and stratification of populations with higher vulnerability to develop AD, as well as aid in the selection of putative targets. Ultimately, this may promote the development of an early and targeted treatment approach during the many decades between the early life environment and clinical AD presentation.

## Box 1. Brain and cognitive reserve hypothesis

The terms brain or cognitive ‘reserve’ have been used as theories to explain individual differences in one’s capacity to maintain cognitive function despite the emergence of brain pathology and individual differences in pathology [116, 211]. For example, some individuals (with a possible increased brain or cognitive reserve) can tolerate more pathological alterations than others before functional deficits appear [107]. The underlying neurobiological mechanisms behind why one person develops AD symptoms later than another person, with comparable pathology, remains elusive, but several possibilities have been proposed, mainly in relation to resilience of the brain to AD neuropathology [212].

Possible explanations for a later development of clinical symptoms of AD include: enhanced resistance of the brain to withstand the effects of disease causing agents (e.g. by more efficient or more effective cellular defence mechanisms and detoxification or clearance mechanisms); better compensatory or repair responses to an insult; a higher level of brain ‘plasticity’ and capacity to functionally adapt in general; and larger numbers of synaptic connections or neuronal numbers [213, 214].

Producing a large *cognitive reserve* is in the first place thought to be instrumental in delaying later disease onset. Cognitive reserve has been linked to functional adaptations and a large extent of ‘flexibility’ in the adult and aging brain, which may have been installed as a result of positive stimulation of the brain during sensitive early life periods—for example, by growing up in an enriched and intellectually more stimulating environment, or by receiving more years of education and/or mental training or challenges [214]. This has also been phrased as ‘use it or lose it’ [179]. As defined here, *brain reserve* is a related concept, generally referring to differences in neural substrates, like brain size, neuron numbers, synapses, or dendritic complexity, that may to some extent be driven by genetic factors but that can also be modified by early life experiences [215].

## Box 2. Rodent models of AD neuropathology

Preclinical studies have employed transgenic and non-transgenic approaches to model aspects of Alzheimer’s disease. These models generally reproduce various disease aspects: memory impairments, Aβ-containing plaques and/or tau/tangles, and neuronal loss (only in a few Aβ-based models).

Transgenic models most frequently (over)express single or multiple mutations in the APP, presenilin (PS), and/or tau genes, or combinations of these genes, that relate to familial forms of AD. Non-transgenic models are generated by the injection of specific toxins into the brain, such as Aβ, tau, or inflammatory-related compounds, or use naturalistic models of aging. Although none of these models fully captures the entire human disease profile and they often model only one specific aspect of AD neuropathology, the existing models have made important contributions to our current understanding of AD pathophysiology. There are, however, distinct differences in the presentation of neuropathology in transgenic models and the human presentation of dementia, in particular with regard to animal models of amyloid pathology which overall display severe hippocampal amyloidosis, which is different from the human presentation of plaque pathology. Also, no tau mutations have been identified that cause autosomal dominant AD, unlike mutations in Aβ-associated genes. The Aβ and tau-based models will be discussed here in more detail.

*Aβ neuropathology*. The amyloidogenic pathway of amyloid precursor protein (APP) processing occurs through APP cleavage by β and γ-secretases, producing C83, C99, and Aβ fragments. Aβ peptides can aggregate to form oligomers, which exist in different forms (e.g. soluble/insoluble, oligomeric, fibrillary plaques) and have different pathogenic properties. The most commonly used mouse models overexpress a mutant form of APP (isoform 695) with the Swedish mutation (KM670/671NL) (‘Tg2576’ mice), resulting in elevated levels of Aβ and cognitive impairments by 1 year of age [216]. The introduction of an additional PSEN1 mutation, which increases γ-secretase activity, yields the widely used APPswe/PS1dE9 mouse, which develops progressive Aβ deposits and cognitive impairments as early as 6 months [217, 218].

*Tau neuropathology*. Tau proteins are the product of the microtubule-associated protein tau (MAPT) gene, and mutations in this gene lead to hyperphosphorylated Tau. Excessive levels of this protein, or its abnormal phosphorylation, both result in the formation of NFTs and pathogenic paired-helical filament tau. The PS19 [219], Tau.P301L [220], and JNPL3 [221] models overexpress the MAPTP301L gene, and show progressive tangle-like pathology in the midbrain and brain stem, parallel to cognitive deficits (not reported in JNPL3 mice). Given the preferential targeting of the disease gene to these brain regions and the important role of tau for (large) motor neurons, many tau mutant mice develop motor problems prior to the onset of hippocampal and cognitive impairments, which is a drawback of these models.

*Combined neuropathology*. When multiple transgenes are combined, both Aβ and tau neuropathology is induced, for instance in the bi-genic model overexpression APPV717I and Tau.P301L mutation (‘biAT’) [222]. Other commonly used models are the 3xTg-AD, harbouring the APP Swedish, the MAPT P301L, as well as the PSEN1 M146 V mutations, displaying learning deficits from 6 months onwards [223]. The 5xFAD model, harbouring the APP Swedish, Florida, and London mutations, as well as the PSEN1 M146 V and PSEN1 L286 V mutations show aggressive and early presentation of amyloid pathology, starting at 1.5 months of age [224]. Additional and related models have also been generated [226, 227].

## Box 3. Rodent models of early life stress and enhancement

In the early life period, the brain shows massive development and is highly sensitive to environmental factors that can disturb this process and affect brain function for life. The effects of environmental factors depend on the maturity of the brain at the moment of intervention.

In animal models, the critical components shaping the local environment are the intrauterine environment (that can be affected by specific medication or, for example, stress hormones that reach the pregnant dam and her fetus(es)) and postnatal experiences. In rodents, the most relevant factor during the early postnatal period involves interaction between the dam and her offspring. This includes elements like tactile stimulation, nutrition, and warmth. Both prenatal and postnatal time windows can be manipulated experimentally to study the consequences of early life experiences.

First, models are used in which the *naturally occurring variation in maternal care* is used to select for pups that received high amounts of maternal care compared to pups receiving low amounts of maternal care (*low vs high licking and grooming*). This represents a model to test the consequences of ‘negative’ and stressful versus a ‘positive’ early life environment for later brain structure and function [139, 229].

*Prenatal stress* [211] encompasses stress induced in pregnant rodents by a single or repeated session of maternal restraint stress and/or defeat during specific gestational periods (mostly during the last week of gestation, sometimes earlier).

Alterations in the postnatal mother–pup interaction can also be induced experimentally. *Postnatally*, early life stress is, for example, induced by a single, prolonged separation of dam and pups (*maternal deprivation* [142]), which usually lasts for 24 h and is conducted at postnatal day (PND) 3 or 4. Alternatively, *maternal separation* [230] involves separation of the dam and pups repeatedly for 2–5 h/day. *Chronic early life stress* [231] involves a reduction in the available nesting and bedding material, which triggers erratic and fragmented maternal care and stress in the dam which is transmitted to her offspring.

In contrast, a ‘positive’ early life environment is typically installed by separating the dam and her pups for a brief period of up to 15 min on a daily basis, during a time window from PND 2 to 9 or until weaning. This model is generally called postnatal or *neonatal handling* [232–234] and results in increased levels of maternal care of the dam towards her pups upon reunion.

## Box 4. Outstanding questions

**Rodent studies**

1. *How does early life adversity enhance the vulnerability to develop AD pathology?*

Early life adversity regulates AD pathology later in life. Although there is evidence that the time of onset and/or severity is affected, an important question remains regarding which mechanisms are involved. This requires a deeper understanding of the role of environmental factors altering Aβ production (e.g. early changes in HPA axis activity) and clearance (BBB, neuroimmune response), but also on molecular factors (REST, EGR1) that determine synaptic function and the sensitivity of synapses for Aβ.

(2) *When is the brain most sensitive to factors that determine the later vulnerability to develop AD pathology?*

Studies on early life adversity and AD have mostly focused on different prenatal and postnatal periods (until weaning), while effects of stress during adolescence and adulthood have also been reported. A critical question is what are the actual most critical time windows during which the brain is most sensitive for early life adversity and later sensitivity to develop AD pathology?

(3) *Does early adversity affect cognitive reserve?*

There are ample indications that early life stress affects brain reserve. However, whether and how early life adversity affects cognitive reserve remains to be determined in more detail. To this end, how cognitive reserved is defined neurobiologically and mechanistically is imperative in order to converge findings from the rodent and human literature. In particular, it is critical to understand the underlying neuronal networks, connections, and synaptic properties that mediate cognitive reserve. Behaviourally, it will be important to understand whether (and how) early life adversity affects learning strategies and behavioural flexibility in AD mouse models, as well as measures of cognitive reserve.

(4) *Can the resistance of the brain to develop AD pathology be enhanced?*

While the questions already mentioned focus on the consequences of early life adversity, it will be of equal importance to determine whether and how cognitive stimulation and/or early life enrichment can reduce the sensitivity for AD pathology. Is it possible to enhance neuronal activity and promote plasticity in relevant brain areas in order to delay AD-related neuropathology and cognitive decline? This includes studies on the developmental trajectories of AD pathology, its mechanisms, and sensitive time windows. In line with this, it will be important to investigate whether effects of early life adversity on the sensitivity to develop AD pathology can be prevented or normalised. This may involve factors such as exercise, cognitive stimulation, nutrition, and/or pharmaceutical intervention.

**Human studies**

1. *Do early life experiences affect AD in humans?*

Rodent studies indicate a strong relationship between early life experiences and the development of AD pathology. It remains elusive whether such associations are also found in humans. Can existing human longitudinal cohort studies confirm the associations found preclinically between early life experiences, AD vulnerability/resilience, and alterations in brain function and cognition?

(2) *What are the critical time windows for development of AD pathology?*

Can critical time windows be identified in humans during which stress modifies AD risk? Which are the critical periods for early stress in humans, and can interventions during those periods indeed interfere with the effects of early adversity on later AD changes?

(3) *Is it possible to modify vulnerability for AD pathology?*

It will not only be important to understand whether and how effects of early life adversity can be overcome, but in general whether and how strategies recruited to increase the resistance for developing and delaying AD pathology can be optimised and implemented. Based on fundamental studies, this may involve strategies such as cognitive stimulation, exercise, and nutrition.

## Abbreviations

AD: Alzheimer’s disease
ApoE4: Apolipoprotein E4
APP: Amyloid precursor protein
Arc: Activity regulated cytoskeleton-associated protein
Aβ: Amyloid beta
BACE1: β-APP cleaving enzyme 1
BBB: Blood–brain barrier
biAT: Bigenic APPswe and Tau.P301L
CA1–3: Cornu ammonis 1–3
CRH: Corticotropin releasing hormone
CSDS: Chronic social defeat stress
EGR1: Early growth response protein 1
FAD: Familial Alzheimer’s disease
GC: Glucocorticoid hormone
GR: Glucocorticoid receptor
HPA: Hypothalamic–pituitary–adrenal
IDE: Insulin-degrading enzyme
LTP: Long-term potentiation
MAPT: Microtubule-associated protein tau
MCI: Mild cognitive impairment
NFT: Neurofibrillary tangle
PFC: Prefrontal cortex
PND: Postnatal day
PS: Presenilin
REST: Repressor element-1 silencing transcription factor
